# Why mobile health app overload drives us crazy, and how to restore the sanity

**DOI:** 10.1186/1472-6947-13-23

**Published:** 2013-02-11

**Authors:** Lex van Velsen, Desirée JMA Beaujean, Julia EWC van Gemert-Pijnen

**Affiliations:** 1 National Institute for Public Health and the Environment, P.O. box 1, Bilthoven, 3720, BA, the Netherlands; 2 Department of Psychology, Health & Technology, University of Twente, P.O. box 217, Enschede, 7500, AE, the Netherlands

**Keywords:** Health apps, Smartphone, Tablet PC, Open source, Standardization, Personalization

## Abstract

**Background:**

Smartphones and tablet computers have become an integral part of our lives. One of their key features is the possibility of installing third-party apps. These apps can be very helpful for improving health and healthcare. However, medical professionals and citizens are currently being overloaded with health apps. Consequently, they will have difficulty with finding the right app, and information and features are fragmented over too many apps, thereby limiting their usefulness.

**Discussion:**

In order to combat health app overload, suppliers of apps need to do three things. One, join the open source movement, so that a few apps can work as gateway to medical information by incorporating information from different sources. Two, standardize content, so that the information provided via apps is readable. And third, in order to prevent information overload from occurring within an app, content should be personalized towards an individual’s characteristics and context.

**Summary:**

Suppliers of medical information and features need to join the open source movement and must make use of standardized medical information formats, in order to allow third parties to create valuable, mobile gateway apps. This can prevent the occurrence of health app overload. By going along in these trends, we can make health apps achieve the impact on healthcare quality and citizens’ health many of us envision.

## Background

### The rise of mobile technology

In the last couple of years, we have witnessed a skyrocketing in the sales and use of smartphones and tablet computers. Apple, for example, has sold 35.1 million iPhones and 11.8 million iPads in the second quarter of 2012, representing a growth of respectively 88% and 151% compared to the same quarter a year before [1]. These numbers are part of a trend that shows an increase of sales since the introduction of these devices. The increasing pervasiveness of mobile technology and the speed with which this growth has taken place, has affected the way in which we live our lives: we are online everywhere and anytime [2]. In the end, this development has ensured that mobile technology is now an integral part of daily life, and here to stay.

One of the key features of these mobile technologies is the possibility of installing apps. Apps are software applications that run on smartphones or tablet computers and are distributed via services like the iTunes store (for iPhone and iPad apps) or Google Play (for Android apps). These apps can be authored by the developers of the mobile technology, or by other individuals or organizations, the so-called ‘third-party apps’. Examples of popular third-party apps are the game Angry birds, or the CNN app which provides the latest news.

## Apps for health

Apps have also entered the medical field. In a recent review of articles discussing the development and evaluation of smartphone applications for health, Mosa, Yoo and Sheets [3] make a distinction between apps for healthcare professionals (including disease diagnosis apps, drug reference apps, and medical calculator apps), apps for medical and nursing students (including anatomy tools and electronic versions of medical books), and apps for patients (including chronic disease management apps and fall detection apps). For medical professionals, the use of mobile technology has been found to be beneficial, as it allows them to make decisions more rapidly and with a lower error rate, and to increase the quality of data management and data accessibility [4]. For patients, mobile technology improves patient education, self-management of chronic diseases and it greatly enhances the possibilities for remote monitoring of patients [3]. And these technologies are widely used. A recent study by the Pew Research Center pointed out that 31% of cellphone owners used it to access health information, while 19% of the smartphone owners have installed an app to manage their health [5]. A study among medical providers showed that 56% of them use apps in their clinical practice [6].

Besides the benefits, health apps also bring along a set of challenges. First, the hardware of smartphones poses constraints on usability, such as reading from a small screen, slow download speed, and troublesome input mechanisms [7]. For the case of tablet computers, these issues do not hold [8]. Second, the content of health apps is not always 100% reliable, as often it is created by non-expert writers [9]. This issue has led to several initiatives that aim to assess the quality of a health app, such as the health app guidelines of the U.S. Food and Drug Administration. Third, when a wide range of patient information can be easily collected and shared with health professionals, privacy is a delicate issue [10].

## App overload

**Figure 1:**
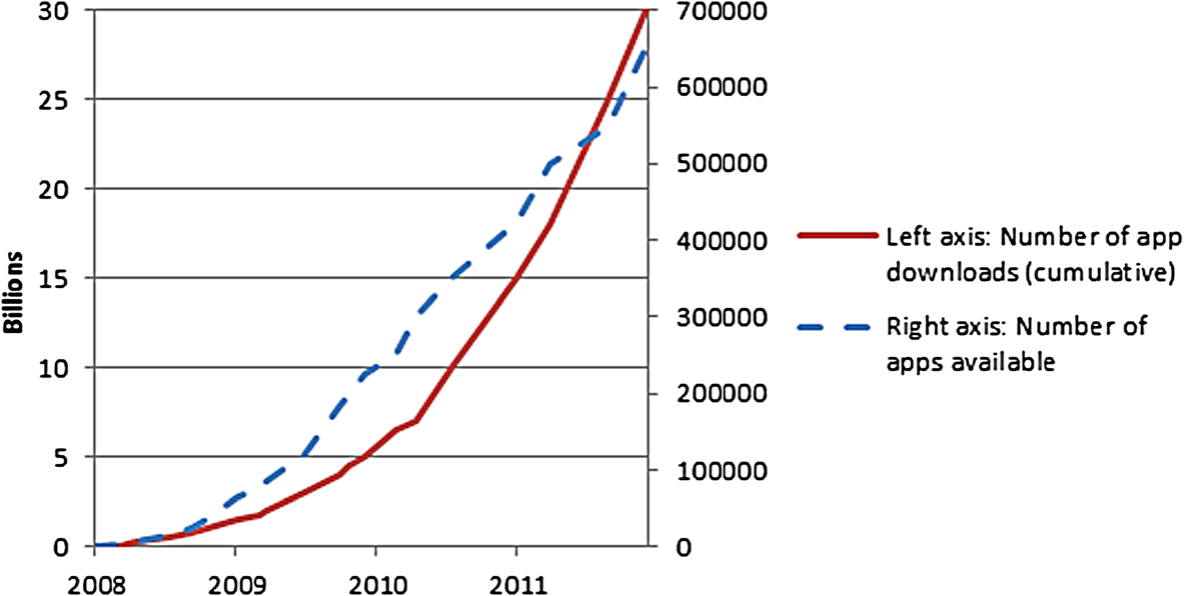
**App downloads and apps offered in the iTunes store (Source: Wikipedia **[11]**).**

Surprisingly, none of the challenges for health apps reported until now, include the great number of apps available for smartphones and tablet computers. As Figure 1 shows, the iTunes store momentarily offers more than 650,000 apps; a number which will continue to grow.

In September 2011, in a piece called ‘The invasion of the mobile apps’, Gary Anthes worded the consequences strikingly: “Today, a stroll through the app stores is a little like visiting an urban flea market, where there are first-rate products but where low-price goods of dubious value abound, and support is practically nonexistent.” [12]. We are witnessing the appearance of the *app overload.* There are too many apps available, and people have difficulty in dealing with the huge supply.

But is app overload also becoming an issue in the health context? According to a study by Epocrates [13], medical professionals have difficulty in finding the right apps for them, while Franko and Tirrell [6] state that physicians may have difficulty in keeping an overview of all the medical apps that are available. Finally, a recent study among medical students and junior doctors in the United Kingdom [14] pointed out that professionals often find medical apps to have limited value, as a separate app does not contain enough material to keep one interested. And during the development of a mobile app that aims to support people in dealing with ticks and tick bites, we interviewed potential users and stumbled upon health app overload among citizens as well [15]. Several of them said they would not download such an app, because they do not want to have so many health apps on their smartphone. So, the consequences of app overload for the health context are:

1. Difficulty in finding the right app

2. Fragmentation of information and/or features over too many apps

Ultimately, this leads to a situation in which the medical professional or citizen cannot find an app, or will not download it as the added value of a single app is too low. And as the supply of health apps is growing, these problems will only get bigger and bigger.

## Discussion

### Tackling health app overload

In order to solve the problems of medical app overload, we must work toward a situation in which a single or a few health apps can serve as gateways to medical information and features. Such a gateway should function as a mobile portal which can lead users directly to the most demanded health content. This should prevent a situation in which a lot of single apps offer help in dealing with single issues like tick bites, burn wounds, making the decision on whether or not to visit their General Practitioner, etcetera. For public health, third-party apps like First aid by the American Red Cross or the mobile version of Microsoft’s HealthVault can serve this purpose; for health professionals, Epocrates is already making progress towards a status as gateway.

**Figure 2:**
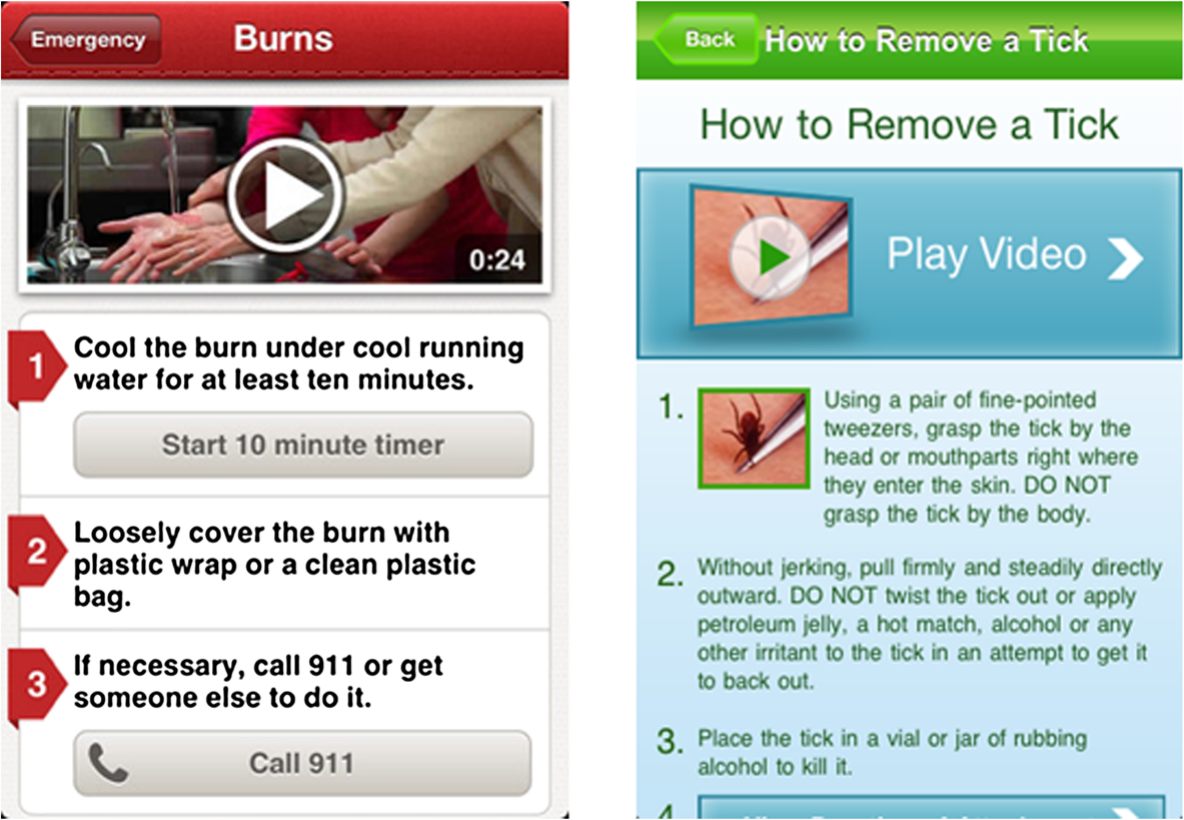
Two sets of instructions on health apps (left: how to treat burn wounds from the First aid app by the American Red Cross; right: how to remove a tick from the Lyme disease tick map app by the American Lyme Disease Foundation).

But in order to create gateway apps, it is crucial that third-party developers gain access to the medical content they need to create high quality health apps. For this they are often dependent on non-commercial health organizations, such as Centers for Disease Control, who create and distribute this content. Therefore, these suppliers of medical information and features must change the way and conditions in which they publish their digital content. They should enable free-market parties to create gateway apps, after which competition among these services is most likely to result in one or a few high quality apps that are most popular. There are two things non-commercial health organizations need to incorporate into their policy:

1. *Join the open source movement*. A gateway health app can only disclose high-quality information and features when it gets access to them. This means that providers of content (Centers for Disease Control, patient associations and hospitals) must allow external parties to use their content and must share technical implementation details under an open source license^a^, instead of only creating their own apps with content no one can reuse. Of course, the suppliers of information and features should be acknowledged by the gateway app, which will also improve its reliability, and will make certification easier. We think that the Center for Disease Control should take an exemplary role in creating and distributing open source content. This may stumble upon certain barriers. Government organizations may be unfamiliar with open source, may not understand it, or may want to protect employment by keeping the development of mobile services within the organization [16]. Therefore, health organizations need to be convinced of the added value of the open source principle, such as working more cost-efficiently [17].In the end they are not giving away their content for free, but are enabling other to reuse it, and are gaining higher reach themselves.

2. *Standardize medical content*. Sharing source data only is not enough. If every supplier employs a different format for communicating medical information and instructions, readability of content provided by the gateway app will suffer. Compare, for example, the instructions for dealing with a burn wound and removing a tick, as displayed in Figure 2. Both are alike in that they first offer a video followed by numbered instructions. But they are also different as the burn instructions only instruct you on what to do, while the tick removal instructions also tell you what *not* to do. App developers and suppliers of information need to agree on a standard for delivering medical information and instructions for mobile devices, and then need to stick to it when producing and publishing it. Such a standard would be the result of an information architecture design process (for an overview of concepts and methods, see [18]). The Center for Disease Control should take a leading role in collaboratively creating and publishing this standard and should be assisted by the U.S. Food and Drug administration, so that the standard complies with quality guidelines.

Once these steps are taken, gateway apps can take a leading role in providing medical information and features to citizens and medical professionals. The next challenge is then to prevent information overload within the app. When all medical content for citizens or professionals is disclosed via just a few apps, chances are that finding one’s way within an app will be difficult. We think the solution here is to personalize the supply of information, instructions and features within an app, based on personal profiles. For example, an 80 year old woman will probably not be interested in information for pregnant women, but will be interested in information about conditions that often affect the elderly, like osteoporosis. By only disclosing the information, instructions and features that align with the characteristics and context of the health app user, information overload can be prevented. There are many techniques available for personalizing apps, such as removing irrelevant text fragments or showing relevant links only [19]. Exactly which technique is best should flow from a thorough context of use analysis. Of course the design of such personalized, mobile health apps should take the specific usability issues for personalization, such as a need for controllability, into account [20].

Finally, there are two developments that can influence the development of app overload in the future. First, the application of guidelines for medical apps by the U.S. Food and Drug Administration^b^ can partly reduce the problem of app overload by regulating app content, thereby making it easier for users to select high quality apps. However, these rules will apply to only a small set of medical apps: those that diagnose and treat disease, and whereby the malfunctioning of the app poses a certain risk [21]. As all those apps focusing on wellness (e.g., an app to help people sleep better) or containing information only will be unaffected, the problem of app overload will not be solved by this regulation. Second, the introduction of mobile Personal Health Record systems (applications that allow patients to record their personal health history themselves) may add to app overload by introducing yet more features, or may diminish app overload as they can also serve as a gateway and set the standards which third party applications need to comply with (for example, Microsoft HealthVault offers a personal health record, but also apps to monitor blood pressure, or to stop smoking). However, for the latter to happen, one mobile Personal Health Record needs to be widely adopted and needs to hold authority in the market. Whether this will actually happen is uncertain, as previous attempts to launch such services have shown that citizens are not eager to use them (the most well-known example being Google Health, which was discontinued due to limited use [22]).

## Summary

In this article, we have expressed our view on the issue of app overload, how this can prevent mobile health apps from being found and used, and how, we think, this issue can be solved: By developing gateway apps that disclose all medical information and features for citizens or medical professionals. For this to happen, suppliers of medical information and features need to join the open source movement and must make use of standardized medical information formats. Finally, gateway health apps need to apply personalization to prevent information overload within the app. We hope that this article will inspire all those who work within the field of health apps to work towards a situation in which app overload is solved by the option to download gateway apps that disclose all relevant medical information, instructions and features for an individual. This way, mobile technology can have the huge impact on healthcare quality and citizens’ health many of us envision.

## Endnotes

^a^ There are many kinds of open source licenses available. An easy and widely used collection of licenses can be found at http://creativecommons.org.

^b^ At the moment of writing, the guidelines for mobile medical apps by the U.S. Food and Drug Administration are under development.

## Competing interests

The author declares that he has no competing interests.

## Authors’ contributions

All authors contributed to the discussion and proposed solutions, discussed in this article. LvV drafted the manuscript. All authors read and approved the final manuscript.

## Pre-publication history

The pre-publication history for this paper can be accessed here:

http://www.biomedcentral.com/1472-6947/13/23/prepub
